# Retrospective study of 124 cases of salivary gland tumors and literature review

**DOI:** 10.4317/jced.55685

**Published:** 2019-11-01

**Authors:** Angélica Reinheimer, Daniella-Serafin-Couto Vieira, Mabel-Mariela-Rodríguez Cordeiro, Elena-Riet-Correa Rivero

**Affiliations:** 1 Postgraduate Program in Dentistry, Federal University of Santa Catarina, Florianópolis, SC, Brazil; 2 Department of Pathology, Federal University of Santa Catarina, Florianópolis, SC, Brazil; 3 Department of Morphological Sciences, Federal University of Santa Catarina, Florianópolis, SC, Brazil

## Abstract

**Background:**

Salivary gland tumors are a rare and morphologically diverse group of lesions and their frequency is still unknown in several parts of the world. The knowledge of its population characteristics contributes to a better understanding of its etiopathogenesis. Objectives: This study investigated the frequency of salivary gland tumors in a region of southern Brazil and compared these data in a literature review.

**Material and Methods:**

A retrospective study was conducted of salivary gland tumors diagnosed at two pathology centers from 1995 to 2016. Patient age and gender, tumor site and frequency, histopathological diagnosis, and symptomatology were evaluated. Chi-squared tests were used to assess the associations between variables. To compare our data, we also conducted a literature review of publications in the PubMed and LILACS databases of retrospective studies of salivary gland tumors.

**Results:**

A total of 124 salivary gland tumor cases was identified, 81 (65.3%) of which were classified as benign and 43 (34.6%) as malignant. Most tumors occurred in the parotid gland (57.2%). Pleomorphic adenoma was the most common tumor (59.6%), followed by adenocarcinoma not otherwise specified (8.8%). The tumors occurred more often in women (54.8%) than in men (45.2%). Malignant tumors were associated with pain in 31.4% of cases (*p*<0.05). The literature review included 35 articles from different countries. Women were most affected, with a mean age of 41.7 years. The most common benign tumor was pleomorphic adenoma (48.2%) and the most common malignant tumor was mucoepidermoid carcinoma (8.7%).

**Conclusions:**

The results of the present study showed that salivary gland tumors are rare. The parotid gland is the most common location and pleomorphic adenoma are the most frequent lesions. The malignant tumors presented as several histological types and the incidence was variable globally.

** Key words:**Salivary gland neoplasms, salivary gland diseases, oral surgery, epidemiology.

## Introduction

A variety of tumors can develop in the salivary glands. Currently recognized 10 subtypes of benign and 20 subtypes of malignant salivary gland tumors (SGT) (1). However, SGT are rare, representing less than 3% all head and neck tumors (2). The majority of SGT are benign, with pleomorphic adenoma (PA) the most common. Among malignant tumors, mucoepidermoid carcinoma (MEC) and cystic adenoid carcinoma (ACC) are more common (2).

Epidemiological studies across the world have shown differences in the incidence and distributions of SGT, with diverse demographic results in different regions (3-4). However, there are few studies about the incidence in the Brazilian population, especially considering its geographical size (2,5-9).

Therefore, the aim of the present study was to investigate the pattern of the occurrence of SGT diagnosed in two diagnostic services that are references to the Santa Catarina State, Brazil and to compare the incidence findings with those available from other places around the world through a literature review.

## Material and Methods

-Retrospective study

The samples included this study were selected from histopathological reports of the Pathological Anatomy Service and the Oral Pathology Laboratory, two diagnostic services at Federal University of Santa Catarina. All cases of SGT diagnosed between 1995 and 2016 were selected. Slides stained with hematoxylin and eosin (H&E) were analyzed by light microscopy (Olympus Corporation, Tokyo, Japan) and classified according to their histopathological characteristics. This study was approved by the University’s Human Research, Ethical Committee (number 1.657.413).

Clinical data were collected from biopsy reports from both pathological services as well as from the university hospital records. The patient information included age and gender. Lesion-related data included histological types, anatomic sites, and symptomatology. Data were collected and stored in a Microsoft Excel® (Microsoft Corporation, Redmond, USA) spreadsheet. Statistical analysis was performed with all collected data using the chi-squared test in the SPSS statistics software to analyze associations between the variables of interest (age, gender, histological diagnoses, and symptoms. The statistical significance was set at α = 0.05.

-Literature review

A review of the literature with no restriction on publication year was carried out to retrieve studies about SGT. The inclusion criteria were retrospective and case series studies without restriction on SGT site or histological type. The exclusion criteria were studies in languages other than English, Portuguese, or Spanish, studies without available full texts and not indexed for PubMed-Medline. An electronic search was performed in the Latin America and Caribbean Health Sciences (LILACS) and PubMed (including MEDLINE) databases on February 2018. The following combinations of keywords were used in the search: “Salivary Gland Neoplasms” OR “Cancer of the Salivary Gland” OR “Salivary Gland Cancer” OR “Salivary gland diseases” OR “Salivary gland tumours” OR “Salivary gland tumors” AND “Retrospective study. All references were managed, and duplicate hits were removed by reference manager software (Endnote X7, Thompson Reuters, New York, New York, USA). The selection of the studies was performed in two phases. In phase 1, titles and abstracts that met the eligibility criteria were selected. If either provided insufficient information for a decision on inclusion/exclusion, the full text was obtained and assessed in phase 2. Those that met the eligibility criteria were also included.

## Results

-Retrospective study

**Figure 1:**
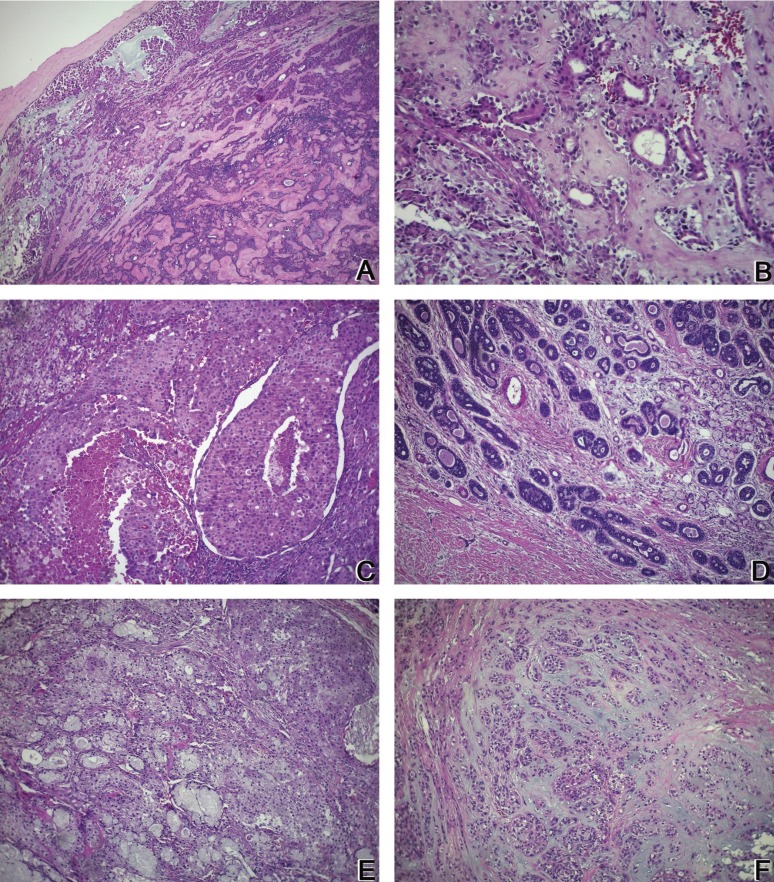
A: Pleomorphic adenoma: (H&E 100x) Tumor composed of epithelial and myoepithelial cells arranged in diverse patterns, surrounded by a fibrous capsule. B: Pleomorphic adenoma: (H&E 400x) Plasmacytoid myoepithelial cells and ductal structures in a fibrous stroma. C: Adenocarcinoma NOS: (H&E 200x) Several cell types, including clear, mucous, and epidermoid cells arranged in different shapes, showing intense cellular and nuclear pleomorphisms. D: Cystic adenoid carcinoma: (H&E 200x) Islands of basaloid epithelial cells containing multiple cylindrical spaces such as cystic spaces filled with basophilic mucoid material. E: Mucoepidermoid carcinoma: (H&E 200x) Nests of pleomorphic epidermoid cells and mucous-producing cells surrounding cystic spaces. F: Carcinoma ex-pleomorphic adenoma: (H&E 200x) Epithelial cells with pleomorphic nuclei arranged in the islands and ductal structures associated with suggestive pleomorphic adenoma areas.

The Pathological Anatomy Service received 126,508 cases for diagnosis between 1995-2016, 103 of which were diagnosed as SGT (0.08%). The Oral Pathology Laboratory received 2,703 cases between 2006-2016, 21 of which were diagnosed as SGT (0.77%). In total, 124 SGT were identified in the two pathology services during the 21-year period. Of the 124 tumors, 81 (65.3%) were benign and 43 (34.6%) were malignant. Of the benign tumors, pleomorphic adenoma (PA) was the most common (91.3%). Of the malignant tumors, adenocarcinoma not otherwise specified (NOS) was the most common (25.5%), followed by ACC, MEC, and carcinoma ex pleomorphic adenoma (Ex-PA) (8 cases each, 18.6%).  Table 1 shows the relative frequencies of the 124 tumors. The histological characteristics of the lesions most prevalent in this study are shown in figure 1.

**Table 1:**
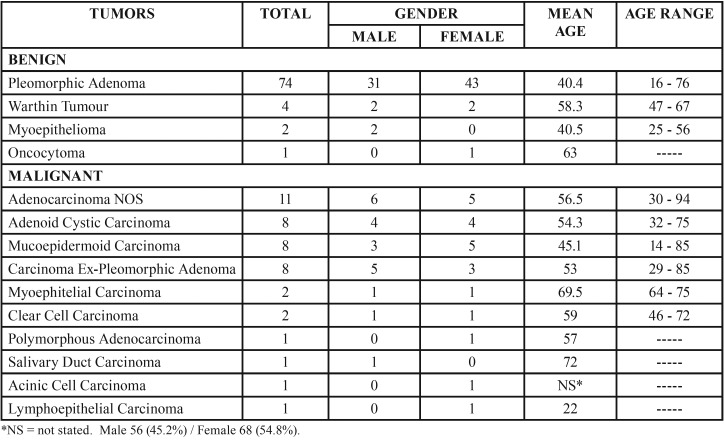
Comparative incidence, gender, and mean age distributions according to the histological subtypes of 81 benign and 43 malignant salivary gland tumors.

The most common site was the parotid gland (57.2%), followed by the minor (29%), submandibular (11.2%), and sublingual glands (2.41%). Regarding the benign tumors, 48 occurred in the parotid gland, 22 in the minor salivary glands, and 11 in the submandibular gland, while 23, 14, and three malignant tumors were identified in the same locations, respectively. Only malignant tumors affected the sublingual glands, with three cases. Among the minor SGT, the palate was the most common site, accounting for 17.7% of all cases, followed by the lip (5.6%) ( Table 2).

**Table 2:**
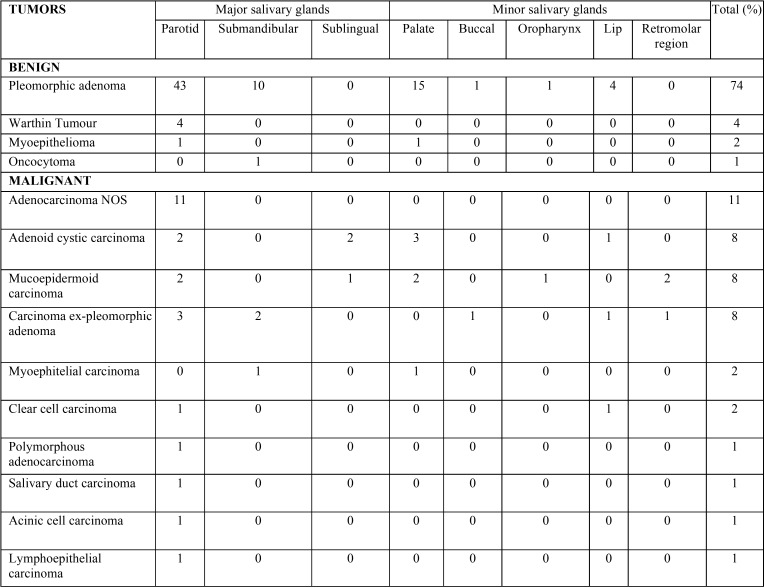
Histological classifications and sites of benign and malignant salivary gland tumors.

Information on symptoms was present for 60 cases of benign tumors; of these, 13 presented pain (21.6%) and one with facial paralysis (1.6%). Among malignant tumors, information was available for 35 cases, 11 of which presented pain (31.4%) and two presented facial paralysis (5.7%). The remaining cases had no symptoms. The chi-square test showed a significant association between malignant tumors and pain (*p* = 0.04).

Patient age was available in 117 cases. The ages ranged from 14 to 94 years ( Table 1). Among benign tumors, the average was 41.3 years, with the highest prevalence in the third decade. Among malignant tumors, the patient average was 54.3 years, with a higher prevalence in the fifth decade. The difference in the numbers of patients with benign and malignant tumors was not statistically significant (*p* = 0.13).

Regarding the distributions by gender, 54.8% and 45.2% of all neoplasms occurred in women and men, respectively (*p* = 0.13). Among benign tumors, 56.7% occurred in women and 43.2% in men (*p* = 0.27). Among malignant tumors, 51.1% occurred in women and 48.8% in men (*p* = 0.12) ( Table 1).

-Literature Review

The literature review yielded 907 citations from the electronic databases. The inclusion and exclusion criteria were applied during a comprehensive evaluation of titles and abstracts, resulting in the selection of 35 studies for full-text review. Based on these studies, the global incidence of SGT was estimated. Fifteen studies were conducted in Asia, eight in Africa, eight in America, three in Europe, and there was one multicenter study in Europe and Asia.

The incidence of salivary gland tumors is higher in women (55.4%) than that in men (47.2%). Considering only the studies that were performed in adults, the mean ages of patients with benign and malignant tumors were 43.3 and 51 years, respectively. Benign tumors (61.9%) were more prevalent than malignant tumors (22.4%), and PA had the highest incidence (48.2%).

Tumors were more common in the major salivary glands (73.2%), especially the parotid gland (58.2%). The minor salivary glands were involved in 24.8% of the cases, most commonly the palate (24.5%). The incidence of the tumors is summarized in  Table 3,  Table 3 continue.

**Table 3:**
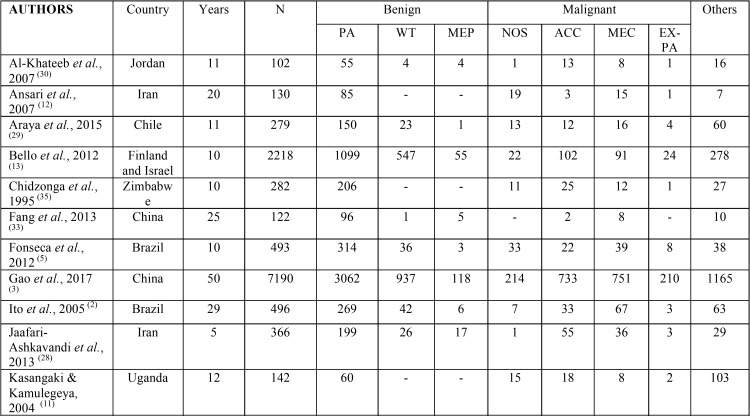
Comparative prevalence of benign and malignant salivary gland tumors in published studies.

**Table 3 continue:**
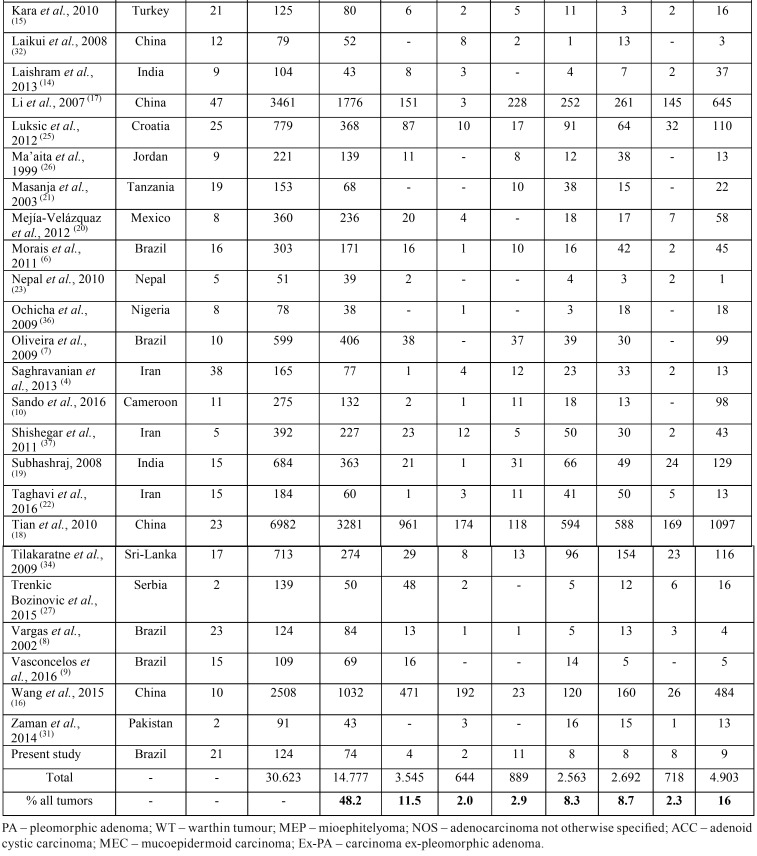
Comparative prevalence of benign and malignant salivary gland tumors in published studies.

## Discussion

During this 21-year study, SGT were found in patients between the ages of 14 to 94 years (mean 43.3). In the current study, patients with benign tumors generally were younger (mean 41.3 years) than patients with malignant tumors (mean 54.3 years). Our data were very similar to those in other studies in Brazil (2,7). The overall mean ages in our review were 43.3 and 51 years for benign and malignant tumors, respectively. Studies from Africa reported lower mean ages of impairment (less than 40 years), suggesting that factors such as low life expectancy and lack of prevention measures may contribute to this index (10-11).

SGT were more common in women (54.8%) than in men (45.2%). However, the distribution of malignant tumors was similar between women and men. Most studies have shown that SGT are more common in women than men, with an average of 55.4% (4,12-14). In contrast, some studies have reported a predominance of SGT in men (15). Two large studies from China has shown that benign tumors are more common in women, while malignant tumors are more common in men (16,17).

In the present study, benign tumors were more common than malignant ones, with frequencies of 65.3% and 34.6%, respectively. This result is similar to the rates reported by studies from China (3,18), India (19), and Mexico (20), suggesting that benign tumors are predominant in SGT worldwide. Brazilian studies reported averages of 80% benign tumors and 20% malignant tumors (7-9). However, in the literature review, the global incidence was 61.9% for benign tumors and 22.4% for malignant tumors. In general, there appear to be geographical variables and the proportions of benign/malignant tumors can be variable. As expected, studies from hospitals reference in oncology report balanced frequency of malignant and benign tumors or more frequency of malignant tumors (21,22).

The majority of SGT occurred in major salivary glands (70%), especially in the parotid gland, similar to average found in the literature (73,2%). Several large series, especially in Asian countries, have shown similar distribution, with more frequency in the parotid, followed by the minor and submandibular gland (3,18,23). Other studies have a higher distribution in submandibular glands than in minor salivary glands (8,12).

The sublingual gland is rarely affected. In the present survey, only three cases were recorded in this location; all were malignant, with two ACC and one MEC. The classification of head and neck tumors of World Health Organization (2005) shows that 70-90% of tumors in sublingual glands are malignant (24). Other studies have also reported a low prevalence and a predominance of malignant tumors in the sublingual glands (16,22,25).

Although most SGT occur in the major salivary glands, the preferred localization of MEC in the present study was the minor salivary glands, contrary to previous studies (6,17,25). ACC occurred in equal frequencies in the major and minor glands. However, some studies have shown ACC to be more common in the minor salivary glands than in the submandibular and parotid glands (2,3,18,26,27).

Considering only the minor salivary glands, the palate was the most frequent location for both benign and malignant tumors. The literature data corroborate this finding (19,28-31). All the studies in the literature review reported the palate to be a common intraoral site. In the present survey, the retromolar region was affected only by malignant tumors. Other studies also show a predominance of malignant tumors in this region (3,32).

PA was the most common tumor (59.6%) of all salivary tumors, accounting for 91.3% of benign tumors in the present study. The frequency of PA among all SGT in the literature ranged from 32.6 to 78.6% (22,33). The average in our literature review was 48.2%. The second most common benign tumor was Warthin’s tumor (4.9%). This agrees with previously published reports, in which the incidence was similar (5% and 4.1%) (19,34). However, its frequency varies between studies, ranging from 0.5% to 18% of all tumors (18,22). The average in our literature review was 11.2%.

Among malignant tumors, adenocarcinoma NOS was the most frequent (25%). The most common location of this tumor, as demonstrated in this study, it is the parotid gland, which is involved in more than 50% of cases (3). In addition, their frequency varies greatly in the literature, due to complex definition. However, it is not usually the most frequent tumor, as demonstrated by our literature review, in which adenocarcinoma NOS comprised only 2.9% of all cases (12,17,25).

In this study, MEC, ACC, and Ex-PA presented at the same frequencies. However, in our literature review, they presented at frequencies of 8.7%, 8.3%, and 2.3%, respectively. The frequency of Ex-PA in the literature is lower than that in our study, showing indexes of about 0.4 to 10% (13,17,20,35). Considering only malignant lesions, some studies reported MEC to be most common (5,26,36), while others reported ACC to be the most prevalent (13,21,27,37).

The most significant sign of benign SGT is a painless swelling. Pain, rapid growth, and an ulcerative surface are noted in malignant cases and especially high-grade tumor (22). In this study, 21.6% of patients with benign tumors reported pain and one patient reported facial nerve paralysis. Among patients with malignant tumors, 31.4% reported pain and 5.7% reported facial nerve paralysis. The pain was significantly more present in malignant tumors (*p* <0.05). This finding agrees with that of Comoglu *et al.*, 2018, who showed that all patients with SGT with preoperative facial paralysis were diagnosed with malignant tumors, suggesting that this sign is indicative of malignancy (38). However, another study reported that three patients with benign tumors presented preoperative facial paralysis due to the compression caused by the lesion; therefore, this symptom should not be considered a sign of malignancy (39).

Studying SGT is difficult because they are a large and diverse group of lesions characterized by morphological heterogeneity (1). Moreover, studies on the relative frequencies of SGT from different parts of the world are difficult to compare because many are based on outdated classification, the number of cases is often small, and the origin of the study differ (medical or dental centers); in addition, the morphological criteria for various histopathological diagnoses vary by pathologist, due in part to their individual training and experience (17,25).

## Conclusions

In summary, the results of the present study suggest that benign tumors are most common, especially in parotid glands, and are represented mainly by PA. Women are the most often affected sex, especially for benign lesions. Regarding malignant tumors, the incidence by gender was variable, as was the incidence of several types worldwide. The findings of this study contribute significantly to the knowledge regarding the incidence of SGT.
